# African swine fever virus: Virology, pathogenesis, clinical impact, and global control strategies

**DOI:** 10.14202/vetworld.2025.1599-1613

**Published:** 2025-06-16

**Authors:** Tridiganita Intan Solikhah, Firda Rostiani, Assyuria Fahma Putri Nanra, Adilah Dwi Putri Paras Dewi, Putri Haibah Nurbadri, Qurrotul Aini Dwi Agustin, Gahastanira Permata Solikhah

**Affiliations:** 1 Department of Health and Life Sciences, Division of Veterinary Clinic, Faculty of Health, Medicine, and Life Sciences, Universitas Airlangga, Banyuwangi, Indonesia; 2 Cahaya Pet Clinic, Veterinarian, Mojokerto, Indonesia

**Keywords:** African swine fever virus, control strategies, epidemiology and transmission, pathogenesis, swine

## Abstract

African swine fever (ASF) is a highly contagious and lethal viral disease affecting domestic pigs and wild boars, with profound implications for global swine production and food security. Caused by the ASF virus (ASFV), a complex double-stranded DNA virus of the *Asfarviridae* family, the disease exhibits diverse clinical outcomes - from peracute death to chronic infection - depending on viral genotype and host immunity. ASFV primarily targets monocytes and macrophages, leading to severe lymphoid depletion, systemic inflammation, and vascular pathology mediated by cytokine storms. The virus demonstrates remarkable environmental resilience and is transmitted through direct contact, fomites, and biological vectors such as *Ornithodoros* soft ticks. With 23 genotypes identified to date, ASFV poses ongoing challenges to diagnosis, control, and vaccine development. Diagnostic methods, including polymerase chain reaction, enzyme-linked immunosorbent assay, and virus isolation, are essential for timely detection and containment. Despite advances in live-attenuated vaccine research, safe and broadly protective vaccines remain elusive. This review synthesizes current knowledge on ASFV’s molecular biology, transmission dynamics, immunopathogenesis, clinical presentations, and control strategies and underscores the urgent need for integrated surveillance systems, cross-sectoral collaboration, and innovative tools for outbreak prediction and disease mitigation.

## INTRODUCTION

Domestic pigs and wild suids are highly susceptible to African swine fever (ASF), a contagious and sev-ere hemorrhagic disease of significant veterinary conc-ern [1]. The virus poses a critical threat to the swine industry and global socioeconomic stability due to its high morbidity and mortality rates, the enforcement of stamping-out policies, restrictions on pork trade and movement, and the lack of effective vaccines and treatments [2, 3]. Although ASF does not possess zoonotic potential [4], its impact on international food security and trade regulations is substantial [3]. Owing to its exceptionally high mortality rate, the spread of ASF is notifiable to the World Organization for Animal Health. Clinical manifestations occur exclusively in domestic pigs and wild boars, while only members of the *Suidae* family are susceptible to ASF virus (ASFV) infection. Among these, warthogs (*Phacochoerus aethi-opicus*) and bushpigs (*Potamochoerus porcus*) serve as asymptomatic carriers and long-term reservoirs of the virus [5].

Despite being first identified nearly a century ago, ASF continues to evade effective disease control due to the absence of curative treatments or broadly protective vaccines. Containment efforts rely primarily on early detection and stringent implementation of conventional disease control measures, including surveillance, epidemiological investigations, animal movement tracing, culling of infected herds, rigorous quarantine protocols, enhanced biosecurity, and move-ment control. Efficient surveillance requires adequate laboratory infrastructure to facilitate rapid diagnostics. When integrated with epidemiological data, such diagnostics enable early disease detection, which is critical for limiting or halting the transmission of ASFV. Although humoral responses are pivotal in diagnosis, passive immunization with anti-ASFV antibodies has been shown to reduce viremia, delay clinical symptom onset, and mitigate disease severity [6]. In addition, cellular immune responses - particularly the activity of natural killer cells and CD8+ lymphocytes - play a crucial role in host resistance against ASFV infection [7, 8]. Cross-protection has also been demonstrated follo-wing the administration of homologous ASFV isolates, some of which are characterized as low-virulence strains [9–11].

Beyond understanding the virological and clinical features of ASF, successful disease mitigation requires the application of predictive modeling to forecast outbreak dynamics and assess the socioeconomic outcomes of interventions such as isolation, culling, safe carcass disposal, and movement restrictions. These strategies are particularly critical in low- and middle-income countries, where ASF outbreaks can significantly compromise livelihoods and food security [12].

Although numerous studies have described the virology, pathogenesis, and epidemiology of ASFV, there remains a lack of an integrative, up-to-date synthesis that bridges molecular mechanisms, host immune responses, diagnostic advancements, and practical disease control strategies, especially in the context of evolving outbreaks in low- and middle-income countries. Furthermore, limited attention has been given to translating immunological and genomic insights into predictive models and field-applicable mitigation tools, which are urgently needed for resource-constrained settings.

This review aims to provide a comprehensive and multidisciplinary overview of ASFV, encompassing its molecular virology, pathogenesis, clinical features, diagnostic approaches, immune responses, and cont-rol strategies. By synthesizing current evidence and highlighting recent advances, the review seeks to support the development of effective surveillance systems, inform policy and biosecurity practices, and identify research directions that facilitate vaccine devel-opment and predictive outbreak modeling.

## CAUSATIVE AGENT

ASFV is a large and complex double-stranded DNA virus classified under the Asfarviridae family and *Asfiv-irus genus* [13]. Genotypic analysis based on the *p72* gene has identified 23 distinct ASFV genotypes; however, only a single serotype has been conclusively defined, underscoring the complexity of immune protection against the virus [14]. Despite the existence of a single serotype, recent studies have proposed the division of ASFV into eight serogroups based on hemadsorption inhibition (HAI) tests in tissue culture. Unlike classical swine fever (CSF) virus, ASFV is distinguished by its capacity for hemadsorption. Genomic variation exists among isolates, with genome sizes ranging from 170 to 190 kilobase pairs and encoding 151–167 open reading frames [15].

Structurally, ASFV exhibits a multilayered icosahedral morphology, with virus particles approxi-mately 200 nm in diameter composed of about 50 proteins [16]. However, cryo-electron microscopy studies on field isolates from China have revealed particle sizes ranging from 260 to 300 nm [17]. During infection, ASFV expresses over 150 proteins, many of which are highly immunogenic and contribute to viral replication and immune evasion [18]. Notably, nearly 50% of ASFV-encoded genes remain funct-ionally uncharacterized, presenting a major barrier to elucidating the virus’s pathogenesis and immuno-mod-ulatory mechanisms [19].

Monocytes and macrophages are the primary target cells for ASFV in both domestic and wild suids [20]. Although lymphocytes are not directly infected, they undergo apoptosis as a consequence of viral infection [21]. ASFV is also capable of replicating in hepatocytes, renal tubular epithelial cells, neutrophils, and endothelial cells [22]. The variability in disease manifestation is attributed to differential viral replication dynamics and host immune responses. Soft ticks of the genus *Ornithodoros* – particularly *Ornithodoros moubata* in Africa and *Ornithodoros erraticus* in the Iberian Peninsula - are integral to the epidemiological cycles of ASF and act as biological vectors for ASFV replication [23].

The majority of ASFV replication occurs in the cytoplasm, although the initial stages of viral DNA synthesis are nuclear [24]. ASFV replication is biphasic: Following early nuclear replication, viral DNA is amplified in cytoplasmic virus factories adjacent to the nucleus [25]. While transcription and DNA replication are virus-encoded, translation relies on host cellular machinery [26].

Due to its reliance on arthropod vectors, ASFV is unique among DNA viruses in being classified as an arthropod-borne virus (arbovirus) [27]. The virus can persist for up to 5 years in *Ornithodoros* ticks [28]. While all domestic pig breeds and age groups are susceptible, wild African pigs serve as natural reservoirs. In addition, emerging evidence suggests that leeches may act as alternative reservoirs for ASFV [29]. The virus preferentially targets cells of the monon-uclear phagocyte system, particularly monocytes and macrophages [17].

The mechanisms by which ASFV enters host cells remain incompletely understood. The current evidence suggests that macropinocytosis and clathrin-mediated endocytosis are involved in viral entry. However, the viral ligands and host cell receptors implicated in this process have yet to be fully characterized. After internalization, ASFV particles are trafficked to the perinuclear region through the endosomal pathway, where acidification promotes uncoating and release of the viral genome into the cytoplasm [30].

ASFV is remarkably resilient in the environment and resists physical treatments, including freezing, thawing, ultrasonic disruption, and cold storage. It remains infectious for extended periods at low tempe-ratures: Up to 65 weeks at −20°C and several years at −80°C. Thermal inactivation requires heating to 56°C for 70 min or 90°C for 30 min. The virus can persist for up to 182 days in raw or undercooked pork and marinated products and up to 105 days in decomposing pig carcasses [31].

Advances in functional genomics and reverse genetics have begun to elucidate the roles of previously uncharacterized *ASFV* genes in virulence, host specificity, and immune modulation. One such gene, H240R, has emerged as a critical virulence determinant. Its deletion results in enhanced NOD-like receptor protein 3 (NLRP3) inflammasome activation, rendering it a potential target for the development of live-attenuated vaccines [32].

## EPIDEMIOLOGY OF ASF

ASFVs exhibit a wide host range within the *Suidae* family, including domestic pigs, European wild boars, bush pigs, and warthogs [33]. Direct transmission of ASFV occurs when susceptible animals come into close proximity to infected animals or ingest or inhale virus-laden secretions and environmental contaminants [34]. Indirect transmission may also occur through biting insects, human-mediated activities, contaminated livestock vehicles, fomites, or tainted food products. *Ornithodoros* soft ticks function as both biological vectors and long-term reservoirs, thereby facilitating ASFV persistence in endemic areas [35].

Infected domestic pigs and wild boars excrete the virus in all bodily fluids and excretions, such as urine, feces, and oronasal secretions. Notably, virus shedding begins approximately 2 days before the onset of clinical signs. Because viral concentration is highest in blood, environmental contamination is exacerbated by hemorrhaging and, in some cases, bloody diarrhea in affected animals [36].

The first documented ASF outbreak outside the African continent occurred in 1957 near Lisbon, Portugal. The outbreak was traced to pigs that had consumed raw swill containing infected pork from planes arriving from Angola [37]. In 2018, ASFV re-emerged in domestic pig populations across several countries, including the Russian Federation, China, Vietnam, and South Korea, demonstrating its potential for rapid transcontinental dissemination [38–40]. Surveillance efforts have confir-med ASFV antibodies in wild boars in the European Union [41] and Estonia [42].

An increasing number of reports from ASF-endemic regions describe circulating ASFV strains with variable virulence, resulting in diverse clinical prese-ntations among susceptible animals. ASFV exhibits genetic variation in several key genes, contributing to the emergence of novel genotypes. Phylogenetic analysis using the World Organization for Animal Health (WOAH)-recommended polymerase chain reaction (PCR) targeting the *P54* and *P72* genes is routinely applied for molecular epidemiological investigations of ASFV [43].

Genotype II was likely introduced into Madagascar through Mozambique [44, 45]. The ASFV strain detected in Mauritius belonged to the same phylogenetic cluster as genotype II viruses previously isolated in Madagascar and Mozambique between 1998 and 2007. In contrast, genotype I has been consistently identified in all West African isolates [44] as well as in sylvatic hosts from East and Southern Africa [46]. Until the recent introduction of genotype II into the Caucasus and Russia, genotype I was the only lineage identified outside the African continent. Molecular analyses suggest that East Africa was the most probable origin of the ASFV strain intro-duced into Georgia [47].

In response to the initial ASF outbreak, Portuguese veterinary authorities enforced strict quarantine protocols, conducted mass depopulation of infected herds, sanitized affected pig farms, and imposed a mandatory restocking delay. Initially, pig movement was restricted to designated “sanitary control zones,” but commercial pressures weakened enforcement, allowing subsequent outbreaks in other regions. Despite this, the disease was successfully contained by veterinary services in 1958, and no new cases were reported in Portugal for the following 2 years [37].

Environmental variables, particularly rainfall and temperature, significantly influence ASFV survi-vability and transmission. Annual average temperatures and precipitation levels, especially during the driest months are critical determinants of environmental virus persistence. With ongoing climate change, the expansion of suitable habitats may facilitate the global spread of ASFV [48]. In addition, high wild boar popu-lation densities increase transmission risk through contact with infected carcasses and direct interactions. Seasonal variations and hunting activities can modify wild boar movement patterns, thereby elevating ASFV transmission risks in regions beyond Africa [49].

## CLINICAL FEATURES OF ASF

ASF manifests variably in wild and domestic pigs, with clinical outcomes ranging from peracute death to chronic forms, depending on the virulence of the virus and the host’s immune status [50]. The incubation period following infection with a virulent strain ranges from 2 to 7 days, occasionally extending up to 14 days. Clinical signs caused by highly virulent ASFV strains include high fever, loss of appetite, dyspnea, cutaneous hyperemia, and inactivity. Other symptoms may involve elevated body temperature, anorexia, conjunctival inflammation, vomiting, diarrhea (ranging from watery to profuse), tachypnea, tachycardia, miscarriage in pregnant sows, anemia, and incoordination. In severe cases, acute forms with hemorrhagic manifestations such as petechiae and epistaxis have been observed.

### Classification of clinical forms by virulence

ASFV isolates are categorized into highly virulent, moderately virulent, and low virulent strains. Accor-dingly, clinical courses in domestic pigs are described as hyperacute, acute, subacute, or chronic. While highly virulent strains typically cause peracute or acute disease, moderately virulent isolates may lead to acute, subacute, or even chronic forms [51].

### Peracute and acute forms

**Figure 1:**
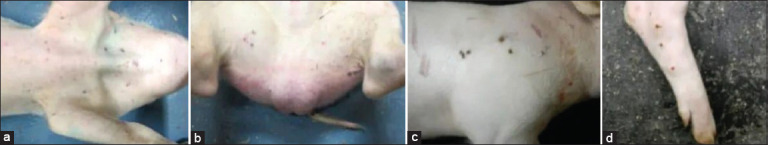
Animals suffering from subacute African swine fever may have skin and skeletal muscle lesions. (a) ecchymoses and petechial hemorrhages in the chest and neck, (b) ecchymoses and petechial hemorrhages in the belly, scrotal sac, and caudal skin areas, (c) focal skin hemorrhages on the lateral thorax, and (d) petechial hemorrhages on the skin of the hind limb. All figures are adopted from Vidana *et al.* [52].

Highly virulent ASFV strains induce peracute or acute ASF. Acute cases may also arise from moderately virulent strains. Peracute ASF is extremely severe, progresses rapidly, and often leads to sudden death, sometimes without preceding clinical signs. Acute ASF is characterized by abrupt fever (40.5°C–42°C), anorexia, respiratory distress, and generalized weakness. Affected animals may group together, lie recumbent, and exhibit reluctance to move (Figure 1a) [52]. Pregnant sows may abort due to febrile episodes, which are often among the first signs of an outbreak [53]. Additional signs in acute cases include coughing, mucopurulent nasal discharge, elevated respiratory and heart rates, epistaxis (Figure 1b), dyspnea, and ocular secretions (Figure 1c). Digestive disturbances such as diarrhea, constipation, vomiting, and abdominal pain may occur. Diarrhea may range from mucoid to bloody and can result in melena, contaminating the perineal area.

**Figure 2:**
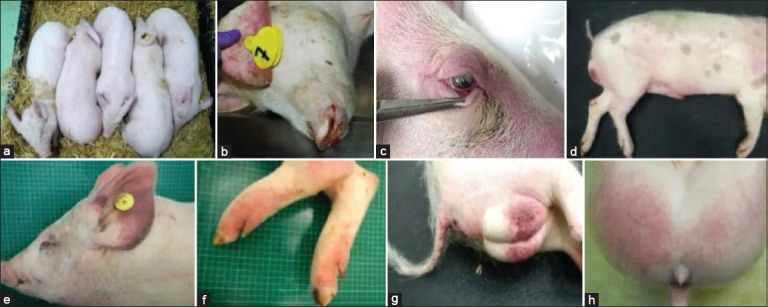
External lesions and clinical symptoms in animals suffering from acute African swine fever. (a) Sluggish pigs huddled together, (b) nasal hemorrhages (epistaxis) and ear skin erythema, (c) ocular discharge and congested ocular mucosa, (d) cutaneous erythema and cyanosis in the ears, neck, dorsal and lateral skin areas, abdomen, and scrotal sac, (e) severe cyanosis and ear hemorrhages, (f) diffuse erythema and petechial hemorrhages on the hind limbs, (g) cyanosis and hemorrhages in the tail, perianal area, and scrotal sac, and (h) erythema and petechial hemorrhages in caudal skin areas. All figures are adopted from Vidana *et al*. [52].

Skin changes include erythema and cyanosis, especially on the ears, nose, abdomen, chest, scrotum, perianal region, and tail (Figures 1b, 1d, and 2d) [52]. Cyanotic patches typically emerge 24–48 h before death. Cutaneous petechiae and ecchymoses are prominent in light-skinned pigs and serve as early diagnostic markers (Figures 2e, g, h). Terminal stages are marked by hypothermia, coma, and death [54, 55]. Differential diagnosis with CSF is particularly difficult during the early stages due to overlapping clinical signs such as pyrexia and hemorrhages.

### Subacute form

Moderately virulent strains can result in subacute ASF, with a clinical course lasting up to 4 weeks. While some pigs may remain asymptomatic, others show milder signs similar to acute ASF, including moderate to high fever and variable mortality (30%–70%). The incubation period ranges from 2 to 7 days, and clinical signs may last 7–20 days. Pyrexia often exceeds 40.5°C and fluctuates irregularly. Abortions in pregnant sows may occur early. Neurological signs are more common and severe in this form. Lesions include purplish discoloration, petechiae, ecchymoses (Figures 2a and b), and necrotic skin patches (Figures 2c and d). Hemorrhagic skin lesions are also observed [18, 54, 55].

### Chronic form

**Figure 3:**
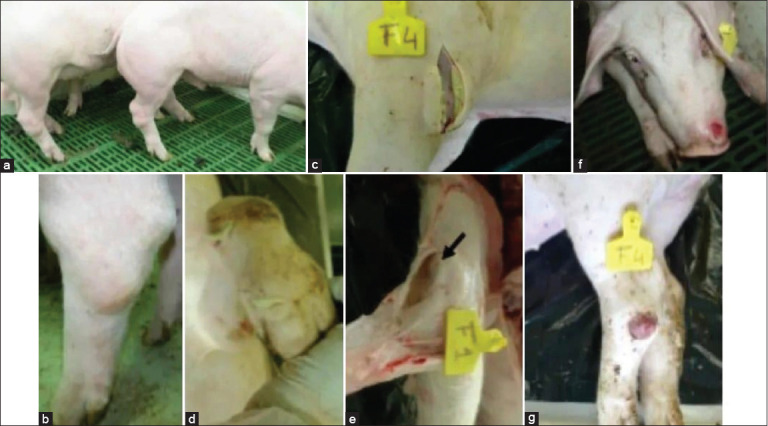
Animals suffering from chronic African swine fever may have lesions in their skeletal muscle, joints, or skin. (a and b) Swelling of the joints, (c) purulent periarthritis, (d) purulent arthritis, (e) serofibrinous periarthritis, (f) skin ulcers in the nose and (g) skin ulcers on the limbs. All figures are adopted from Vidana *et al*. [52].

Chronic ASF is associated with moderately or lowly virulent isolates, often emerging in the later stages of outbreaks. Clinical courses can last over a month, with some cases persisting up to 15 months. Mortality rates are typically below 30%. Signs include intermittent fever, weight loss, emaciation, joint swelling due to arthritis or periarthritis (Figures 3a–e) [52], and occasional lameness. Cutaneous manifestations such as ulcers and necrotic patches are more pronounced than in subacute forms, particularly in areas over bony prominences, ears, nose (Figure 3f), neck, flank, abdomen, and limbs (Figure 3g).

ASFV may persist in the blood or tissues of animals recovering from subacute or chronic forms for up to 3 months, contributing to silent spread and sporadic outbreaks [53, 56].

### Epidemiological implications of chronic ASF

Chronic ASF forms, typically caused by low-virulence isolates, are characterized by non-specific symptoms and may be misdiagnosed as other diseases such as CSF. Infected pigs often exhibit low mortality but show signs of growth retardation, respiratory distress, arthritis, and persistent ulcers. Survivors may become asymptomatic carriers, intermittently shed the virus, and pose a significant risk for undetected transmission. Chronic cases commonly present with multifocal skin necrosis, arthritis, weight loss, and abortion. Unlike acute forms, vascular changes are typically absent. Lesions often result from secondary bacterial infections, including fibrinous polyserositis, necrotic or chronic pneumonia, and necrosis of the tonsils, skin, and tongue. Postmortem findings may include lymphadenopathy, lung necrosis, and occasional pericarditis, though less severe than those in acute infections. The persistence and subtlety of clinical signs in chronic ASF complicate control and eradication efforts [55].

## PATHOGENESIS OF ASF

In Africa, ASFV infection in wild suids - particularly warthogs and bushpigs - can result in mild clinical signs and chronic infections [57]. In contrast, transmission dynamics among domestic pigs are predominantly shaped by human-mediated activities. The movement of personnel, contaminated equipment, transport vehicles, clothing, and feed supplies is a major contributor to the farm-to-farm spread of ASFV. In addition, the virus can persist for extended periods in frozen meat, posing a threat of re-emergence when such meat is used in swill feeding practices [58].

### Entry and initial replication

Infection typically occurs through the oral or nasal routes following direct contact with infected animals or through bites from infected soft ticks [57]. After entry, initial viral replication takes place in the tonsillar and mandibular lymphoid tissues. The virus then disseminates systemically through the lymphohematogenous route [59, 60]. The principal cellular targets of ASFV are mononuclear phagocytic cells, particularly macrophages and monocytes [61]. Early during infection, ASFV actively suppresses apoptosis in infected cells, thereby enhancing viral replication and systemic spread [62].

### Immunopathological effects

ASFV infection induces apoptosis of lymphocytes and impairs macrophage function, resulting in profound immunosuppression and widespread syste-mic inflammation [59]. Replication of the virus in monocytes and macrophages results in the release of proinflammatory cytokines, including tumor necrosis factor-alpha (TNF-α), interleukin (IL)-6, IL-8, and IL-1 [61]. This response mimics a cytokine storm, characterized by elevated levels of TNF-α, IL-1β, and IL-6, which contribute to increased vascular permeability, hemorrhage, and ultimately, multiorgan failure [63, 64]. The pathogenesis of ASFV shares notable similarities with viral hemorrhagic fevers, particularly in terms of cytokine dysregulation, endothelial injury, and coagu-lopathy [65–67].

### Lesions and histopathology

Hemorrhagic or hyperemic splenomegaly is the most consistent gross lesion observed in acute ASFV infections, with severity correlating with the virulence of the infecting strain [68]. Hemorrhagic manifestations are also commonly seen in the skin, kidneys, and lymph nodes, particularly in areas with high macrophage concentrations and minimal smooth muscle tissue. While hemorrhages occur early in infection, endothelial cell involvement generally arises later in the disease course [61].

In lymphoid tissues, such as the spleen and lymph nodes, ASFV infection leads to lymphocyte depletion and necrosis. The bone marrow often exhibits hypocellularity, predominantly affecting the myeloid lineage, and a marked reduction in hematopoietic precursor cells [36, 63, 69]. Although ASFV has systemic effects on immune cell populations, the specific molecular mechanisms responsible for lymphocyte apoptosis, endothelial dysfunction, and cytokine storm generation remain poorly defined. This represents a critical research gap in the comprehensive understanding of ASFV-induced immunopathology [60, 63, 64].

## PATHOLOGICAL FEATURES OF ASF

ASFV is a highly contagious and often fatal pathogen that induces severe pathological alterations in both domestic and wild pigs. One of the most noticeable gross lesions in acute infections caused by highly virulent strains is splenomegaly, which presents as an enlarged, dark-colored, and friable spleen. Hemorrhagic lymphadenitis is commonly observed, particularly in the renal and gastrohepatic lymph nodes [68]. Microscopically, ASFV causes extensive lymphoid depletion and apoptosis, especially in the spleen and lymph nodes. The virus targets mononuclear phagocytic cells, leading to the necrosis of macrophages and a heightened release of proinflammatory cytokines, such as IL-1 and TNF-α. ASFV infection of endothelial cells can exacerbate vascular thrombosis and leakage. In chronic ASF, typically associated with less virulent strains, lymphoid tissues exhibit granulomatous infla-mmation and fibrosis, indicative of a prolonged immune response [25].

Rodríguez-Bertos *et al*. [59] documented consistent gross pathological findings of acute ASFV infection in all animals examined. External assessments revealed congestion and hemorrhaging in ocular mucosa, eyelid edema, and erythematous skin lesions, particularly evident in hairless areas like the abdominal wall and inguinal or axillary regions. Some animals presented with hemoperitoneum, hemothorax, or hemopericardium, indicated by blood clots in these cavities. All animals exhibited mitral valve thickening, edema, and pallor. Lung pathology was significant, especially in the caudal lobes, where the absence of collapsed parenchyma highlighted lobular prominence. Most specimens displayed dilated interlobular septa due to interstitial edema, multiple petechiae, and focal lobular congestion (score 1). The lung surface appeared mottled, with widespread petechiae and ecchymoses that often coalesced to form large hemorrhagic areas. Some animals exhibited bronchointerstitial pneumonia (score 2), evident by depressed areas with pleural adhesions or fibrinous pleuritis. In two cases, 90% of the lung tissue displayed firm, dark red lobular consolidation (score 3) due to severe congestion and hemorrhage.

**Figure 4:**
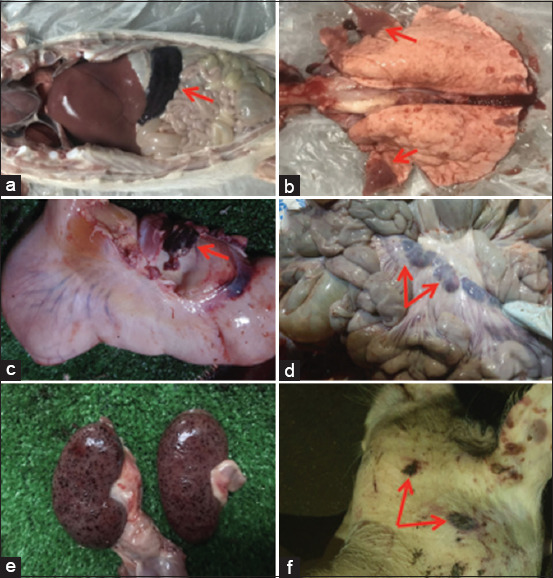
(a) Upon exploration of the abdominal cavity, hemorrhagic splenomegaly (arrow) is visible, (b) multifocal hemorrhages and several regions of lung consolidation in the cranial lobes (arrows), (c) The gastrohepatic lymph node has hemorrhagic lymphadenitis (arrow), (d) mesenteric lymph node hemorrhagic lymphadenitis (arrows), (e) the renal cortex has several large petechial hemorrhages, and (f) multiple localized hemorrhages on the head and neck skin (arrows). All figures are adopted from Nga *et al*. [70].

Nga *et al*. [70] observed that hyperemic or hemo-rrhagic splenomegaly consistently occurred in ASFV-infected animals, with affected spleens appearing enlarged and dark red (Figure 4a). The lungs showed consolidated areas across various lobes, predominantly in the cranial and medial regions, accompanied by multifocal hemorrhages (Figure 4b). Hemorrhagic lymphadenitis was also noted, especially in renal, gastrohepatic, and mesenteric lymph nodes (Figure 4c and 4d). Additional hemorrhages were present in the kidneys, gastrointestinal tract, respiratory system, and externally on the skin (Figure 4e–f).

Although ASFV infection is typically associated with leukopenia and thrombocytopenia, the severity and nature of hematological abnormalities vary depending on the strain’s virulence and the host’s susceptibility. Zhu *et al*. [69] reported that infection with the highly virulent Kirawira isolate (KWH/12), initially described by Greig and Plowright [71], results in decreased leukocyte counts without significantly affecting red blood cell numbers.

## LYMPHOID PATHOGENESIS AND MULTISYSTEM INVOLVEMENT

ASF induces profound pathological alterations in lymphoid organs, including the spleen, lymph nodes, thymus, and tonsils. A hallmark lesion is severe lymphoid depletion marked by apoptosis and necrosis of lymphocytes, particularly in the spleen and lymph nodes [59, 63]. Although lymphocytes are not directly infected by ASFV, their widespread apoptosis results from infection of monocytes and macrophages, which release inflammatory mediators that trigger immune cell death. This leads to generalized immunosuppression and leukopenia, especially lymphopenia.

**Figure 5:**
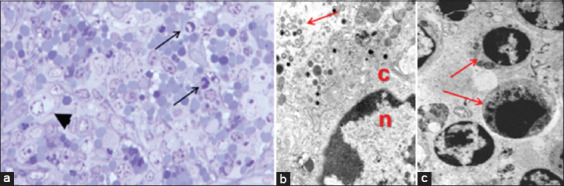
(a) A pig experimentally infected with acute ASF (3 dpi) has a spleen segment that is semithin (1 μm) and stained with toluidine blue. The macrophage has nuclear chromatin margination and a clear juxtanuclear intracytoplasmic inclusion body (arrowhead), (b) transmission electron microscopy picture, which was taken from the spleen of an ASFV-infected pig, demonstrates the margination of nuclear chromatin and the existence of a viral factory in the cytoplasm (arrow) in the macrophage nucleus (n) and cytoplasm (c), and (c) lymphocytes (arrows) in the spleen of a pig experimentally infected with acute ASF (5 dpi) undergo apoptosis. All figures are adopted from Salguero [68]. ASF=African swine fever, ASFV=ASF virus.

Histological analyses reveal depletion of both B and T lymphocytes, follicular necrosis, and infiltration of histiocytes and macrophages containing viral antigens [59, 60]. Initial viral replication begins in the tonsils and mandibular lymph nodes, with systemic dissemination through lymphohematogenous rou-tes occurring within 2–3 days [68]. Bone marrow suppr-ession is also observed, leading to leukopenia and thrombocytopenia [19]. In peripheral blood, ASFV alters immune cell profiles, resulting in marked lymphopenia and monocytopenia, which may be accompanied by neutrophilia as a stress response [71]. Lymphocyte depletion is mediated by bystander apoptosis induced by cytokines such as TNF-α and IL-1β, which also contribute to endothelial injury and increased vascular permeability. These processes exacerbate the loss of immune cells and tissue damage. Spleen enlargement (hemorrhagic splenomegaly), follicular disruption, and thymic atrophy are commonly observed, especially in piglets [69, 72]. Spleen pathology (Figure 5) [68] illustrates the severe impact of ASFV on lymphoid architecture and immune functionality.

This systemic immune dysregulation, driven by cytokine-induced vascular injury, also affects other organs, particularly the lungs. Clinical manifestations include vomiting, bloody diarrhea, hindlimb ataxia, and respiratory difficulty, often resulting from pulmonary edema and leading to mortality. Histologically, necrotic mononuclear cells are evident in alveolar septa and lumina. Vascular endothelial damage in the lungs contributes to alveolar wall thickening and arterial thrombosis [73].

**Figure 6:**
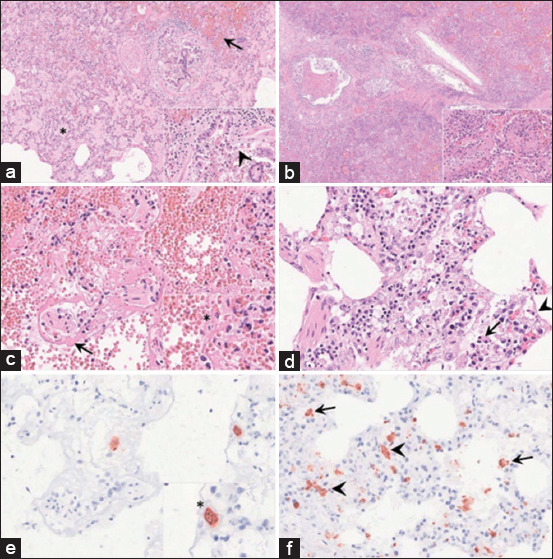
(a) Protein-rich alveolar edema with fibrin strands and erythrocytes, (b) alveoli with severe necrotizing bronchopneumonia, (c) loss of alveolar epithelium and hyaline membrane, (d) inflammatory infiltration and epithelial necrosis, and (e and f) viral antigen-positive macrophages in alveolar regions (immunohistochemistry, anti-p72 antibody). All figures are adopted from Miao *et al*. [74].

Figure 6 [74] illustrates these pulmonary lesions; Figure 6a shows alveolar spaces filled with protein-rich edema, erythrocytes, and fibrin strands, indicating heightened vascular permeability. Bronchioles display epithelial necrosis, erythrocyte infiltration, and cellular debris. Figure 6b presents severe fibrinosuppurative to necrotizing bronchopneumonia, marked by infiltration of neutrophils, plasma cells, macrophages, lymphocytes, and cellular debris. Figure 6c highlights alveolar epithelial loss and hyaline membrane formation. Intravascular macrophages are prominently visible in Figure 6d, which also shows alveolar septal thickening and epithelial necrosis in bronchioles. Immunohistochemical staining for the anti-p72 antigen confirms ASFV-infected macrophages in multiple lung compartments (Figures 6e and f), emphasizing that macrophage-mediated inflammation and cytokine storm are key drivers of lung pathology [59, 60, 74].

### Multiorgan histopathology

**Figure 7:**
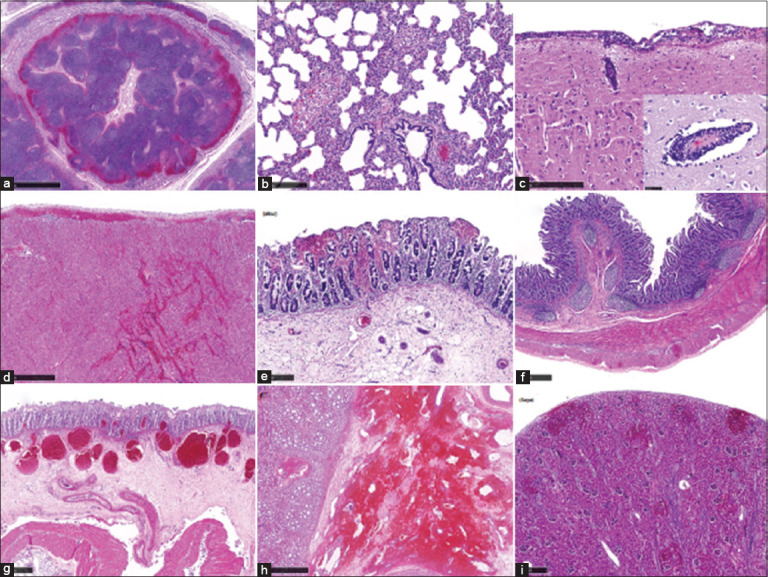
H&E staining of African swine fever-infected pig tissue. (a) Sinus hemorrhage was present in the lymph node tissue of case 9, (b) interstitial pneumonia and increased numbers of pulmonary intravascular macrophages were present in the lung tissue of case 9, (c) three to four layers of mononuclear cell infiltration in the meninges and perivascular cuffing in the cerebral tissue of case 20. The insert shows swollen endothelial cells and apoptotic bodies in the perivascular cuffing lesion, (d) epicardial and myocardial hemorrhages with mononuclear cell infiltration were present in the cardiac tissue of case 9, (e) multifocal hemorrhages were seen in the superficial and profound lamina propria of the gastric epithelial tissue in case 9, (f) multifocal hemorrhages were present in the outer longitudinal muscularis layer of the small intestine tissue of case 9., (g) the colon tissue in case 58 showed multifocal hemorrhage in the inner lamina propria and dilated capillaries filled with erythrocytes in the submucosal layer with extensive edema underneath, (h) there was extensive bleeding in the pelvic region of the renal tissue in case 9, (i) multifocal hemorrhage was found in the cortical area, sometimes extending to the capsular layer of the renal tissue in case 9. All figures are adopted from Izzati *et al*. [60].

According to Izzati *et al*. [60], necrosis, sinus bleeding, apoptotic bodies, and hemorrhagic lesions were commonly observed in lymph nodes (Figure 7a) [60]. Unlike tonsils, the lymph node netw-Vork displayed distinct lymphoid follicles. Interstitial pneumonia was present in all eight lung samples, with mononuclear infiltration elevating interstitial fluid levels (Figure 7b). Pulmonary intravascular macrophages were frequently detected in small capillary vessels, while alveolar macrophages were absent. Pulmonary edema was found in 5 of 8 cases, bleeding in 2 of 8, and congestion in 3 of 8 cases.

In the brain, meningitis (in 8/8 cases) and perivascular cuffing (in 5/8 cases) were observed, with mononuclear cells exhibiting apoptotic features or nuclear fragmentation (Figure 7c). Neutrophils were rarely detected in meningeal tissues. Severe capillary congestion was typical, although hemorrhage was minimal. Perivascular edema was accompanied by endothelial swelling, but glial nodules were absent.

Cardiac tissue analysis revealed epicardial and myocardial hemorrhages in 3/8 cases (Figure 7d), sometimes accompanied by mononuclear inflammatory infiltration. Hemorrhagic lesions were also present in gastrointestinal tissues. Case 58 showed lamina propria bleeding and submucosal edema in gastric tissue (Figure 7e). Case 18 exhibited multifocal hemorrhage in the muscularis layer of the small intestine (Figure 7f), while Case 19 showed inner lamina propria bleeding, capillary congestion, and submucosal edema in the colon (Figure 7g).

Renal lesions were observed in all cases, with bleeding in the renal pelvis (Figure 7h) and edema in Cases 57 and 59. Petechial hemorrhages in the renal cortex were found in 5 of 8 cases, sometimes extending to the capsular surface (Figure 7i). No lesions were noted in the renal medulla. Multifocal interstitial nephritis was diagnosed in 5–8 cases, demonstrating widespread renal involvement in ASFV pathology.

## PREVENTION AND CONTROL STRATEGIES

Preventive strategies for ASF should target the primary modes of pathogen transmission to mitigate the risk of outbreaks in swine production systems. Epidemiological evidence indicates that ingestion of contaminated food (such as swill feeding) and direct physical contact between pigs are major routes for ASFV transmission [9]. Although large commercial pig farms benefit from stringent biosecurity protocols, wild pigs infected with ASFV can contaminate the environment and pose significant epidemiological threats to domestic herds [72].

Contrary to initial assumptions, soft ticks are not primary vectors of ASFV in all epidemiological contexts; rather, the virus is predominantly transmitted through direct contact between infected and susceptible animals or indirectly through contaminated feed, fomites, or personnel [75]. Thus, early detection remains the most effective strategy for ASF control and the preservation of animal health. Field practitioners in the Philippines identified swill feeding, movement of pers-onnel without adequate biosecurity, and inadequate disinfection practices as principal contributors to ASF emergence [76].

Comprehensive laboratory testing of both wild and domestic pig populations is crucial for active and passive surveillance programs aimed at preventing outbreaks and facilitating rapid responses. Such testing supports the identification of infection hotspots and informs the design of effective control measures [77].

To prevent ASFV introduction into uninfected regions, strict import regulations are vital. These include the prohibition of live-infected animal imports and the restriction of contaminated animal products. For control and eradication, key measures involve the prompt culling and disposal of infected animals, imposition of movement restrictions, and enforcement of marketing bans in affected zones [3].

A major advancement in ASF prevention is the development of live attenuated vaccines. One of the first commercially available ASF vaccines, ASFV-G-∆I177L, was developed in Vietnam. This vaccine demonstrated safety, as inoculated animals did not exhibit clinical signs throughout a 28-day observation period, unlike those infected with the virulent Eurasian strain [78]. However, challenges persist due to the virus’s complex structure. ASFV is a large, double-stranded DNA virus (170–190 kb) that encodes approximately 170 proteins, many of which play roles in immune modulation and evasion [79]. Ongoing research is crucial for optimizing vaccine efficacy, ensuring saf-ety, and achieving cross-protective immunity against diverse ASFV genotypes.

## INFECTION AND IMMUNITY

The clinical manifestations of ASFV vary significantly between wild boars and domestic pigs, correlating to a wide spectrum of pathomorphological findings [50]. ASFV targets the cytoplasm of the reticuloendothelial or mononuclear phagocytic system, particularly monocytes and macrophages, which play a critical role in its pathogenesis [80]. The alimentary tract represents the primary natural route of infe-ction; however, alternative routes such as respiratory inhalation, skin abrasions, injections, and insect bites have also been implicated [53]. Upon entry through the tonsils or posterior pharyngeal mucosa - independent of tick vectors - the virus disseminates to the mandibular or retropharyngeal lymph nodes, leading to viremia and systemic distribution. Secondary replication primarily occurs in organs such as the spleen, bone marrow, liver, lungs, kidneys, and endothelial tissues associated with mononuclear phagocyte systems [64].

### Immune targets and protective antigens

The hemagglutinin protein CD2v/EP402R is considered one of the most crucial ASFV antigens associated with protective immunity [81]. This protein, along with the auxiliary viral antigen C-type Lectin/EP153R, plays a pivotal role in serogroup-specific immune responses [82]. CD2v is also among the most variable gene orthologs across ASFV isolates. Notably, p72 genotyping does not consistently correlate with homologous or heterologous cross-protection, as genetically divergent strains may induce cross-protective immunity while closely related strains may not. These findings underscore the novelty and complexity of ASFV serotype-specific protective immunity mediated through HAI mechanisms [15].

### Unresolved aspects of ASFV immune response

The immunological responses following ASFV infection remain incompletely understood, owing to the virus’s sophisticated immunoevasion strategies [83]. Because ASFV replicates in mononuclear phagocytes, it disrupts cytokine homeostasis, leading to coagulopathy and the dysregulated release of pro-inflammatory cytokines [71]. Some researchers suggest that neutralizing antibody responses are weak or absent following ASFV infection; however, others have demonstrated the presence of neutralizing antibodies directed against p32, p54, and p72 proteins [84]. Nevertheless, the protective effects attributed to these proteins have not been reliably replicated across different pathogenic models, casting doubt on the protective efficacy of antibody-mediated responses and prompting further investigation into their role in ASFV immunity [85].

### Cytokine storm and immune dysregulation

Cytokines play a central role in modulating the immune response to pathogenic insults, coordinating both innate and adaptive arms of immunity. However, excessive cytokine production - referred to as a cytokine storm or cytokine release syndrome - can cause systemic inflammation and organ damage [86]. This phenomenon has been observed in various viral infections, including cytomegalovirus, influenza, variola virus, severe acute respiratory syndrome coronavirus 2 (SARS-CoV-2), Middle East respiratory syndrome coronavirus, and SARS-CoV-2 [87]. Given the pathophysiological similarities between ASF and viral hemorrhagic diseases, increased cytokine secretion has been documented in ASFV-infected hosts both *in vivo* and *in vitro* [86].

A prevailing hypothesis posits that ASFV infection in domestic pigs can trigger a systemic cytokine storm, contributing to disease severity and immune-mediated pathology. However, a comprehensive understanding of the kinetics and regulation of cytokine responses in ASFV infection remains lacking [55]. Further research is necessary to elucidate the timing, magnitude, and role of cytokine release in ASFV-induced immunopathology and to guide the development of targeted immunom-odulatory therapies.

## DIAGNOSIS OF ASF

In the absence of effective vaccines or treat-ments, prompt and accurate diagnosis of ASF is a critical control measure. Early and field-deployable detection of ASFV is essential for effective disease monitoring and outbreak management [74].

### Diagnostic samples and core methods

Diagnosis is primarily based on blood and organ tissue samples collected from clinically affected or deceased animals. Confirmatory diagnosis involves molecular and serological techniques, which are indispensable for timely control measures. Core virological tests include PCR-based assays for viral genome detection and antigen detection methods. Other supportive diagnostics encompass direct immunofluorescence, enzyme-linked immunosorbent assay (ELISA), and virus isolation protocols [9].

### ELISA-based antibody detection

The WOAH recommends the use of ELISA for detecting ASFV-specific antibodies. Commercially validated ELISA kits are designed to target prevalent ASFV antigens, including p72, p32, pp62, and p54 [9]. While ELISA offers rapid, cost-effective, and scalable antibody detection, it demonstrates limited sensitivity during the early stages of infection (7–12 days post-infection), necessitating its complementary use with PCR for early-stage diagnosis. However, ELISA is effective for large-scale sero-surveillance due to its capacity to reliably detect antibodies from approximately 12–14 days post-infection [53].

### Virus isolation

Isolation of ASFV from field samples remains a valuable diagnostic approach. When ASFV is present, it replicates in susceptible primary leukocyte cultures, producing characteristic cytopathic effects and hemadsorption reactions. Virus isolation can be performed using porcine blood or alveolar monocyte-derived macrophages. While theoretically feasible for all ASFV strains from natural outbreaks, the method often yields variable results due to sample degradation and the complexity of cultivating primary swine leukocytes [9, 56].

### Emerging molecular technologies

Innovative diagnostic approaches have emerged to enhance the detection of ASFV. A notable advancement by Pepin *et al*. [49] integrated loop-mediated isothermal amplification (LAMP) with the CRISPR/Cas12a system into a single-tube reaction. This method detected as few as 7 copies/μL of the *ASFV p72* gene per assay and demonstrated no cross-reactivity with other swine viruses. The results aligned well with those obtained from real-time quantitative PCR, indicating strong potential for reliable, on-site ASFV detection.

Despite these advances, there remains a significant need for the development and deployment of portable, rapid diagnostic tools suitable for field use, especially in resource-limited settings. Isothermal amplification-based methods and CRISPR-mediated detection platforms hold promise for bridging this gap and enhancing ASFV surveillance capabilities in endemic and at-risk regions.

## CONCLUSION

ASF remains one of the most formidable threats to global swine health due to its complex epidem-iology, lack of effective vaccines, and severe economic consequences. This review consolidates the current understanding of ASFV pathogenesis, immunological responses, clinical manifestations, and diagnostic approaches. The virus targets cells of the mononuclear phagocytic system, primarily monocytes and macrop-hages, triggers lymphoid depletion, systemic cytokine dysregulation, and multi-organ pathology. The most consistent lesions include hemorrhagic splenomegaly, lymphadenitis, pulmonary edema, and widespread vascular injury, all of which contribute to high morbidity and mortality in domestic pigs.

Significant progress has been made in the molecular characterization of ASFV, with over 23 genotypes identified through *p72* gene sequencing. Immunological insights have also improved, particularly regarding CD2v/EP402R and EP153R antigens that contribute to serotype-specific immunity. In addition, field-deployable molecular diagnostics such as PCR, ELISA, LAMP, and CRISPR-based assays offer improved disease surveillance capabilities.

Early detection remains the cornerstone of ASF control, with PCR and ELISA widely used for confirmatory diagnosis and large-scale screening. Effective biosecurity practices, including swill feeding bans, movement control, and disinfection protocols are essential to reduce virus spread. The development of live-attenuated vaccines, particularly ASFV-G-∆I177L, marks a promising step toward immunoprophylaxis in endemic regions.

Despite advancements, several challenges persist. The immunological mechanisms underlying protection and viral clearance remain poorly understood. The inconsistency of cross-protective immunity among ASFV strains, combined with limited field efficacy data for candidate vaccines, hinders the development of effective vaccines. Moreover, diagnostic limitations in resource-limited settings and logistical difficulties in implementing real-time surveillance further constrain disease control efforts.

Future research should prioritize the elucidation of ASFV immune evasion mechanisms and cytokine storm kinetics. The development of broadly protective, genetically stable vaccines suitable for field deployment is urgently needed. Predictive outbreak modeling and geospatial risk mapping can guide targeted control strategies, while improvements in rapid, low-cost, and portable diagnostics will enhance decentralized testing in rural and high-risk areas.

ASFV poses a sustained threat to global food security and the pig industry. An integrated approach – combining molecular diagnostics, targeted vaccination, enhanced surveillance, and stringent biosecurity – is vital to manage and eventually eradicate the disease. A coordinated global effort, particularly in knowledge transfer to low- and middle-income countries, will be crucial in mitigating ASF’s impact and ensuring the sustainability of pig production systems.

## AUTHORS’ CONTRIBUTIONS

TIS, AFPN, ADPPD, and QADA: Drafted and edited the manuscript. PHN and FR: Edited the references. TIS and GPS: Critically revised the manuscript. All authors have read and approved the final manuscript.
